# Supplementary material for: A spatio-temporal analysis investigating completeness and inequalities of global urban building data in OpenStreetMap
Source: Nat Commun. 2023 Jul 6;14:3985. doi: 10.1038/s41467-023-39698-6 (PMC10326063; doi:10.1038/s41467-023-39698-6)
Supplement: Supplementary file 1 — Supplementary Information [file 41467_2023_39698_MOESM1_ESM.pdf]

Supplementary Information – A spatio-temporal analysis investigating completeness and inequalities of global urban building data in OpenStreetMap

Benjamin Herfort[1,2,*], Sven Lautenbach[1], João Porto de Albuquerque[3], Jennings Anderson[4], and Alexander Zipf[1,2]

[1]Heidelberg Institute for Geoinformation Technology, Heidelberg, Germany
[2]GIScience Chair, Institute of Geography, Heidelberg University, Heidelberg, Germany
[3]Urban Big Data Centre, University of Glasgow, United Kingdom
[4]Meta Platforms Inc., Salem, Oregon, USA
[*]benjamin.herfort@heigit.org

ABSTRACT

This file contains the supplementary information.

Supplementary References

1. United Nations Development Programme. *Human development report 2019 : beyond income, beyond averages, beyond today: inequalities in human development in the 21st century.* (2019).
2. OECD. *OECD Regions at a Glance 2016* (2016).
3. Freire, S., MacManus, K., Pesaresi, M., Doxsey-Whitfield, E. & Mills, J. Development of new open and free multi-temporal global population grids at 250 m resolution. *Agile* 6 (2016).
4. Smits, J. & Permanyer, I. Data descriptor: The subnational human development database. *Sci. Data* **6**, 1–15, DOI: 10.1038/sdata.2019.38 (2019).
5. Elvidge, C. D., Zhizhin, M., Ghosh, T., Hsu, F. C. & Taneja, J. Annual time series of global viirs nighttime lights derived from monthly averages: 2012 to 2019. *Remote. Sens.* **13**, 1–14, DOI: 10.3390/rs13050922 (2021).
6. Zanaga, D. *et al.* ESA WorldCover 10 m 2020 v100. Tech. Rep. (2021). DOI: https://doi.org/10.5281/zenodo.5571936.

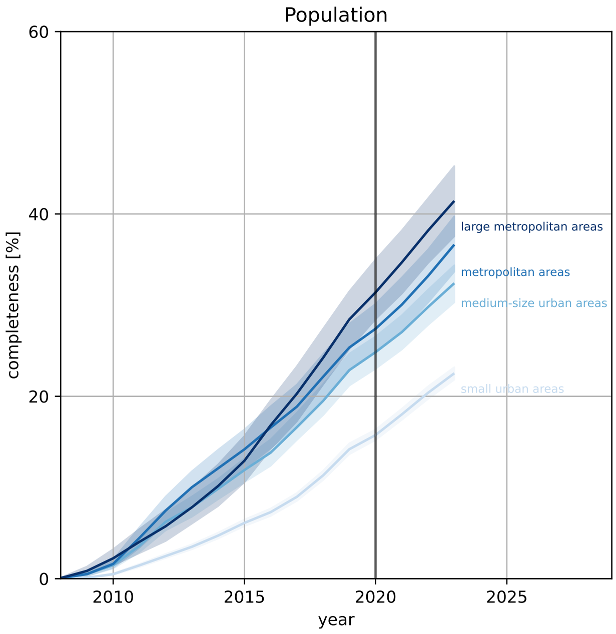

Supplementary Figure 1. Temporal evolution of average urban OSM building completeness by population. Completeness was derived by aggregating building area predictions based on a Random Forests model and monthly OSM building area per urban center. The shaded areas represent the 95% confidence interval for each line. OSM data from 2008-01-01 to 2023-01-01. Created using Matplotlib 3.3. in Python 3.7.5 (https://www.python.org/).

Supplementary Table 1. Precision and recall for the Microsoft building footprints by comparing to Geo-Wiki built-up surface validation dataset. The Geo-Wiki campaign visually assessed very high-resolution satellite images of 50 K sample locations for the presence of built-up surfaces using a crowdsourcing approach. In the Geo-Wiki dataset built-up area is defined as an area containing any building with a roof.

Region	Urban Centers	Geo-Wiki Cells	Recall [%]	Precision [%]
East Asia & Pacific	24	2,352	86.0	87.3
Europe & Central Asia	144	11,531	83.5	86.0
Latin America & Caribbean	91	8,768	83.4	89.8
Middle East & North Africa	55	5,094	84.4	81.7
North America	119	17,373	86.4	88.3
South Asia	70	7,526	84.8	73
Sub-Saharan Africa	161	23,588	81.2	83.3

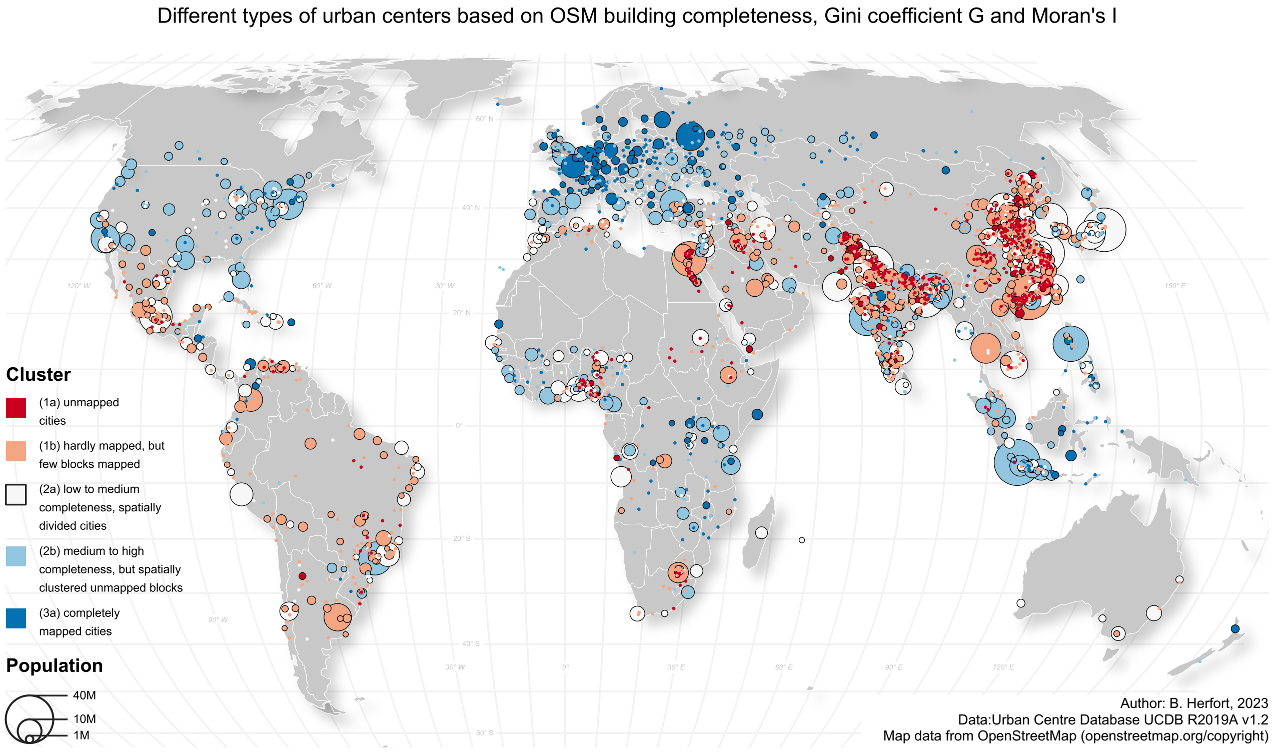

Supplementary Figure 2. Spatial distribution of agglomerative clustering of urban centers based on OSM building completeness, Gini coefficient *G* and Moran's *I*. Each point represents a single urban center with a minimum area of 25 square kilometers (n= 4,647). Smaller urban centers were ignored as Gini coefficient and Moran's I could not be reliably estimated. OSM data as of 2023-01-01. Created using QGIS 3.28.3 (https://www.qgis.org/en/site/).

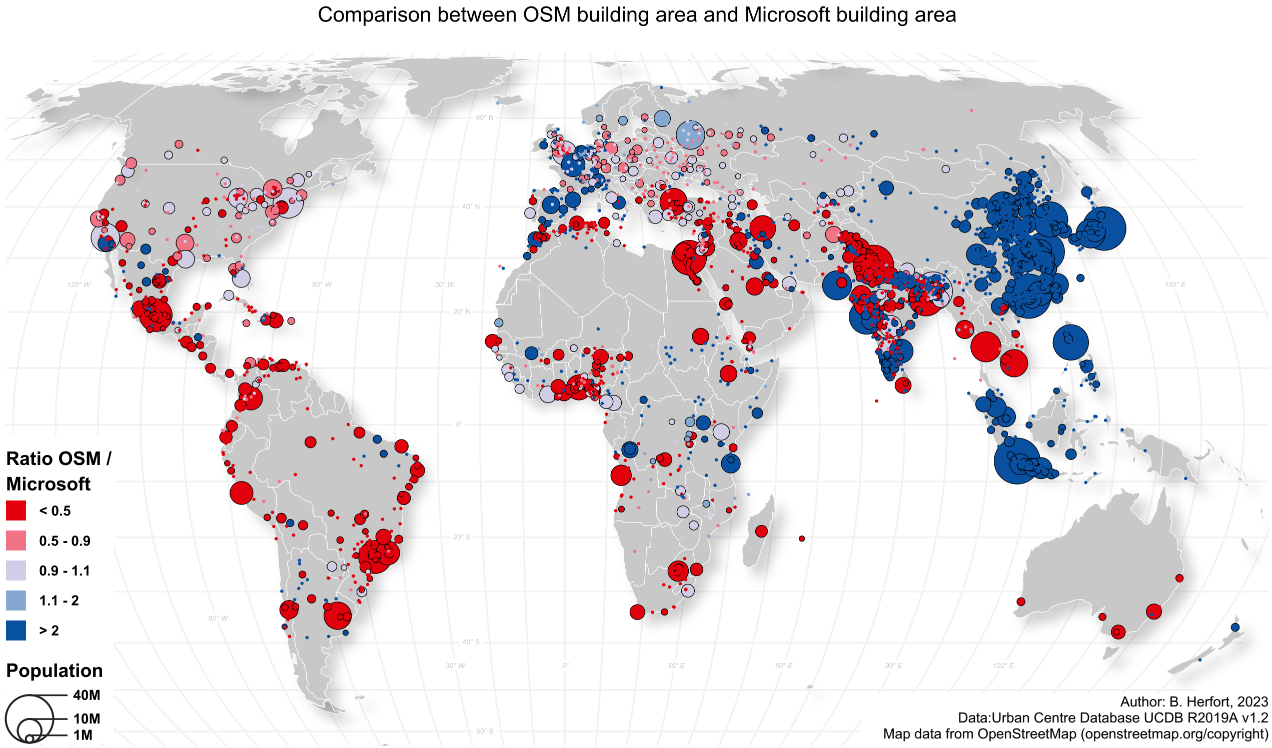

Supplementary Figure 3. Spatial distribution of urban centers based on the ratio between OSM building area and Microsoft building area. This comparison only entails Microsoft building footprints made available through the Global ML Building Footprints dataset as of 2023-01-15. OSM data as of 2023-01-01. Created using QGIS 3.28.3 (https://www.qgis.org/en/site/).

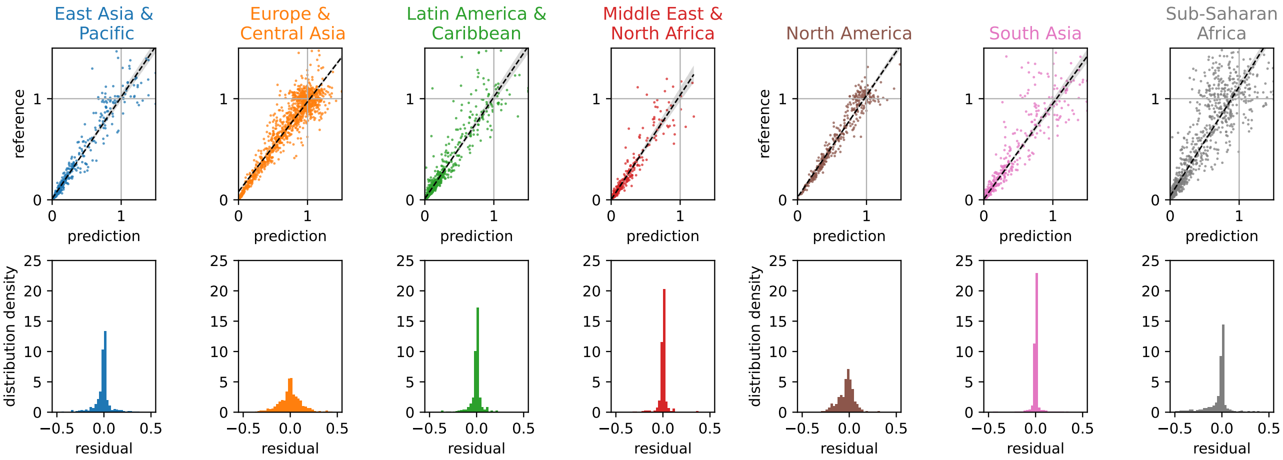

Supplementary Figure 4. Scatterplot of raw residuals in regard to predicted OSM building completeness and distribution density of raw residuals on the urban centers level. The dashed line in the scatter plots depicts the linear regression model fit. The shaded areas represent the 95% confidence interval for the regression estimate. Created using Matplotlib 3.3. in Python 3.7.5 (https://www.python.org/).

Supplementary Table 2. Comparison between OSM building area and Microsoft building area in urban centers on the global scale and grouped by world regions, Subnational Human Development Index class and city size class measured by population. SHDI classes were based on cut-off points defined by the United Nations Development Programme[1]: low human development (SHDI< 0.550), medium human development (SHDI: 0.550 - 0.699), high human development (SHDI: 0.700–0.799), very high human development (SHDI> 0.800). City size classes were based on population thresholds defined by OECD[2]: small urban areas (50k–200k), medium-size urban areas (200k–500k), metropolitan areas (500k–1.5M), large metropolitan areas (>1.5M). This comparison only entails Microsoft building footprints made available through the Global ML Building Footprints dataset as of 2023-01-15. OSM data as of 2023-01-01.

	n	OSM Buildings [sqkm]	Microsoft Buildings [sqkm]	Ratio OSM / Microsoft
Global	13,189	34,531	50,371	0.69
**World Bank Regions**				
East Asia & Pacific	3,068	7,828	2,983	2.62
Europe & Central Asia	1,351	9,839	9,259	1.06
Latin America & Caribbean	1,073	2,198	9,815	0.22
Middle East & North Africa	901	1,156	4,727	0.24
North America	378	9,122	12,347	0.74
South Asia	3,997	1,971	6,366	0.31
Sub-Saharan Africa	2,421	2,417	4,874	0.50
**Subnational Human Development Index**				
Low	2,289	1,109	1,862	0.60
Medium	4,960	4,123	11,519	0.36
High	3,883	7,425	18,122	0.41
Very High	1,967	21,810	24,376	0.89
**City Size by Population**				
Small Urban Areas	10,930	7,151	13,185	0.54
Medium-Size Urban Areas	1,348	4,907	8,018	0.61
Metropolitan Areas	563	6,361	9,931	0.64
Large Metropolitan Areas	287	16,111	24,757	0.65

Supplementary Table 3. Reference datasets used for training the machine learning model.

Dataset Name	Country ISO A3 codes	Urban Centers	Grid Cells
**East Asia & Pacific**			
Microsoft Building Footprints	AUS, KHM, LAO, MMR, MNG, PRK, THA, VNM	332	19,963
GSI Basic Map Information Buildings	JPN	108	22,342
NSDI Continuous Numerical Topographic Map Building Data	KOR	37	5,258
LDS NZ Building Outlines	NZL	8	941
**Europe & Central Asia**			
Microsoft Building Footprints	ALB, ARM, AUT, AZE, BEL, BGR, BIH, BLR, CHE, CYP, CZE, DEU, DNK, ESP, EST, FIN, FRA, GBR, GEO, GRC, HRV, HUN, IRL, ISL, ITA, KAZ, KGZ, LTU, LUX, LVA, MDA, MKD, MNE, NLD, NOR, POL, PRT, ROU, RUS, SRB, SVK, SVN, SWE, TJK, TKM, TUR, UKR, UZB	1,042	69,954
OS OpenMap Local	GBR	135	10,845
IGN BD TOPO Bâtiments	FRA	71	6,292
GUGiK BDOT10k Budynki	POL	48	3,221
NGR Basisregistraties Adressen en Gebouwen (BAG)	NLD	38	2,687
Hausumringe Nordrhein Westfalen, Sachsen, Berlin	DEU[a]	24	4,203
CUZK Budovy (BU)	CZE	12	717
Estonian Land Board Buildings	EST	2	130
**Latin America & Caribbean**			
Microsoft Building Footprints	ARG, BHS, BLZ, BOL, BRA, BRB, CHL, COL, CRI, CUB, CUW, DOM, ECU, GTM, GUY, HND, HTI, JAM, MEX, NIC, PAN, PER, PRI, PRY, SLV, SUR, TTO, URY, VEN	877	52,135
SP Mapa Digital da Cidade	BRA[a]	3	887
Quito Gobierno Abierto Construcciones	Ecuador[a]	2	340
Construcción. Bogotá D.C	Colombia[a]	1	415
BA Data Tejido Urbano	ARG[a]	1	187
**Middle East & North Africa**			
Microsoft Building Footprints	ARE, DJI, DZA, EGY, IRN, IRQ, ISR, JOR, LBN, LBY, MAR, MLT, OMN, PSE, QAT, SAU, SYR, TUN, YEM	666	31,948
**North America**			
Microsoft Building Footprints	CAN, USA	367	100,210
**South Asia**			
Microsoft Building Footprints	AFG, BGD, BTN, IND, LKA, MDV, NPL, PAK	1,677	56,421
**Sub-Saharan Africa**			
Microsoft Building Footprints	AGO, BDI, BEN, BFA, BWA, CAF, CIV, CMR, COD, COG, COM, ERI, ETH, GAB, GHA, GIN, GMB, GNB, GNQ, KEN, LBR, LSO, MDG, MLI, MOZ, MRT, MUS, MWI, NAM, NER, NGA, RWA, SDN, SEN, SLE, SOM, SSD, SWZ, TCD, TGO, ZAF, ZMB, ZWE	1,449	40,534

[a]Building data did not cover the entire country.

Supplementary Table 4. Precision and recall for the Microsoft building footprints as self-declared by Microsoft in the Global ML Building Footprints GitHub repository. The evaluation metrics are computed on a set of building polygon labels for each region. Values might differ across the region.

Region	Recall [%]	Precision [%]
Africa	70.9	94.4
Caribbean	76.8	92.2
Central Asia	79.5	97.2
Europe	85.9	94.3
Middle East	85.4	95.7
South America	78.0	95.4
South Asia	76.7	94.8

Supplementary Table 5. Summary statistics and the impurity based feature importance (Gini importance) for explanatory variables utilized in the machine learning model. World Bank Region code is a categorical variables, thus summary stats are not reported.

Variable	Min	Mean	Median	SD	Max	Feature Importance
GHS-POP 2020[3]	0.0	4484	2750	6104	615757	0.08
SHDI 2019[4]	0.276	0.77	0.77	0.126	0.985	0.04
Average Night-Time Lights 2020[5]	0	121.375	78.0	231.79	71509	0.03
WorldCover 2020 "built-up" [sqkm][6]	0.0	0.403	0.376	0.27	1.005	0.68
WorldCover 2020 "tree cover" [sqkm][6]	0.0	0.228	0.149	0.228	1.008	0.04
WorldCover 2020 "sparse vegetation" [sqkm][6]	0.0	0.083	0.045	0.108	1.003	0.04
OSM road network length 2023 [km]	0.0	9.106	8.133	6.453	57.635	0.06
World Bank Region Code [categorical]	-	-	-	-	-	0.03

Supplementary Table 6. Aggregated OSM road network completeness estimation per world region based on the mapping saturation approach. Mapping activity in urban centers was classified into a "start", "growth" and "saturation" phase. Saturation indicates that most of the mapping of the road network has been completed. During the growth phase the majority of features have been added.

Region	Start	Growth	Saturation
East Asia & Pacific	0.022	0.665	0.302
Europe & Central Asia	0.003	0.145	0.847
Latin America & Caribbean	0.004	0.238	0.752
Middle East & North Africa	0.102	0.504	0.384
North America	0.013	0.016	0.947
South Asia	0.105	0.553	0.334
Sub-Saharan Africa	0.059	0.478	0.460

Supplementary Table 7. Silhouette Coefficient and Calinski-Harabasz Index for various number of clusters. Higher Silhouette Coefficient and Calinski-Harabasz scores relate to a model with better defined clusters.

clusters	Silhouette Coefficient	Calinski-Harabasz Index
2	0.47	4,903
3	0.46	5,026
4	0.39	4,566
5	0.30	4,393
6	0.29	4,125
7	0.29	3,930
8	0.29	3,789
