# Supplementary material for: A spatio-temporal analysis investigating completeness and inequalities of global urban building data in OpenStreetMap
Source: Nat Commun. 2023 Jul 6;14:3985. doi: 10.1038/s41467-023-39698-6 (PMC10326063; doi:10.1038/s41467-023-39698-6)
Supplement: Supplementary file 3 — Reporting Summary [file 41467_2023_39698_MOESM3_ESM.pdf]

nature portfolio

Corresponding author(s): Benjamin Herfort
Last updated by author(s): May 22, 2023

Reporting Summary

Nature Portfolio wishes to improve the reproducibility of the work that we publish. This form provides structure for consistency and transparency in reporting. For further information on Nature Portfolio policies, see our Editorial Policies and the Editorial Policy Checklist.

Statistics

For all statistical analyses, confirm that the following items are present in the figure legend, table legend, main text, or Methods section.

n/a	Confirmed	
☐	☒	The exact sample size (*n*) for each experimental group/condition, given as a discrete number and unit of measurement
☐	☒	A statement on whether measurements were taken from distinct samples or whether the same sample was measured repeatedly
☒	☐	The statistical test(s) used AND whether they are one- or two-sided<br>*Only common tests should be described solely by name; describe more complex techniques in the Methods section.*
☐	☒	A description of all covariates tested
☒	☐	A description of any assumptions or corrections, such as tests of normality and adjustment for multiple comparisons
☐	☒	A full description of the statistical parameters including central tendency (e.g. means) or other basic estimates (e.g. regression coefficient) AND variation (e.g. standard deviation) or associated estimates of uncertainty (e.g. confidence intervals)
☒	☐	For null hypothesis testing, the test statistic (e.g. *F*, *t*, *r*) with confidence intervals, effect sizes, degrees of freedom and *P* value noted<br>*Give P values as exact values whenever suitable.*
☒	☐	For Bayesian analysis, information on the choice of priors and Markov chain Monte Carlo settings
☒	☐	For hierarchical and complex designs, identification of the appropriate level for tests and full reporting of outcomes
☒	☐	Estimates of effect sizes (e.g. Cohen's *d*, Pearson's *r*), indicating how they were calculated

Our web collection on statistics for biologists contains articles on many of the points above.

Software and code

Policy information about availability of computer code

Data collection	All Python code and Jupyter notebooks necessary to calculate the geospatial statistics, create maps and derive figures are available in this GitHub repository: https://doi.org/10.6084/m9.figshare.22241776.
Data analysis	All Python code and Jupyter notebooks necessary to calculate the geospatial statistics, create maps and derive figures are available in this GitHub repository: https://doi.org/10.6084/m9.figshare.22241776.<br>The analysis was conducted in Python 3.10.6 (https://www.python.org/downloads/) based on the packages scikit-learn 1.2.0 (https://scikit-learn.org/stable/), PySAL 2.7.0. (https://pysal.org/) and ESDA 2.4.3 (https://pysal.org/esda/).<br>Plots were created using QGIS 3.28.3 (https://www.qgis.org/en/site/) and the package Matplotlib 3.6.2 (https://matplotlib.org/) in Python 3.10.6.

For manuscripts utilizing custom algorithms or software that are central to the research but not yet described in published literature, software must be made available to editors and reviewers. We strongly encourage code deposition in a community repository (e.g. GitHub). See the Nature Portfolio guidelines for submitting code & software for further information.

Data

Policy information about availability of data

All manuscripts must include a data availability statement. This statement should provide the following information, where applicable:

- Accession codes, unique identifiers, or web links for publicly available datasets
- A description of any restrictions on data availability
- For clinical datasets or third party data, please ensure that the statement adheres to our policy

The full set of data generated in this study (e.g. for training and running the machine learning model and the final results presented in all figures and maps) have been deposited in the Figshare database under accession code https://www.doi.org/10.6084/m9.figshare.22217038.
The Global Human Settlement Layer Urban Centres data used in this study is available from https://ghsl.jrc.ec.europa.eu/ghs_stat_ucdb2015mt_r2019a.php.
Population information has been obtained from GHS-POP, which is available at https://jeodpp.jrc.ec.europa.eu/ftp/jrc-opendata/GHSL/GHS_POP_GLOBE_R2023A/GHS_POP_E2020_GLOBE_R2023A_54009_1000/V1-0/GHS_POP_E2020_GLOBE_R2023A_54009_1000_V1_0.zip.
Subnational Human Development Index data can be retrieved from https://globaldatalab.org/shdi/download/.
The ESA WorldCover dataset is available at https://zenodo.org/record/5571936.
Information on night-time lights was obtained from https://eogdata.mines.edu/nighttime_light/annual/v20/2020/VNL_v2_npp_2020_global_vcmslcfg_c202101211500.average.tif.gz.
The raw full-history planet OSM data can be downloaded from https://planet.openstreetmap.org/planet/full-history/.

Human research participants

Policy information about studies involving human research participants and Sex and Gender in Research.

Reporting on sex and gender	not applicable
Population characteristics	not applicable
Recruitment	not applicable
Ethics oversight	not applicable

Note that full information on the approval of the study protocol must also be provided in the manuscript.

Field-specific reporting

Please select the one below that is the best fit for your research. If you are not sure, read the appropriate sections before making your selection.

☐ Life sciences ☐ Behavioural & social sciences ☒ Ecological, evolutionary & environmental sciences

For a reference copy of the document with all sections, see nature.com/documents/nr-reporting-summary-flat.pdf

Life sciences study design

All studies must disclose on these points even when the disclosure is negative.

Sample size	not applicable
Data exclusions	not applicable
Replication	not applicable
Randomization	not applicable
Blinding	not applicable

Behavioural & social sciences study design

All studies must disclose on these points even when the disclosure is negative.

Study description	not applicable

Research sample	not applicable
Sampling strategy	not applicable
Data collection	not applicable
Timing	not applicable
Data exclusions	not applicable
Non-participation	not applicable
Randomization	not applicable

Ecological, evolutionary & environmental sciences study design

All studies must disclose on these points even when the disclosure is negative.

Study description	This study proposes a machine-learning regression method based on a Random Forest to assess OSM building completeness within 13,189 urban centers (as defined by the European Commission).<br>We utilize an extensive collection of open building data from commercial and authoritative sources as training data and utilize OSM full-history data for spatio-temporal data analysis on the global scale.<br>The model further relies on information obtained from remote sensing data (land cover, population distribution, night time lights), Subnational Human Development Index (SHDI), and urban road network density as predictors.
Research sample	The data used describes all 13,189 urban centers at global scale (as defined by the European Commission).<br>No sample was taken.<br>Accordingly, urban centers have been characterized as high-density clusters of contiguous grid cells of 1 square kilometer with a density of at least 1500 inhabitants per square kilometer and a minimum population of 50,000.<br>Each urban center was spatially disaggregated using a one square kilometer grid based on the equal-area Mollweide projection.<br>The grid adopted the same structure utilized by the raster datasets of the GHS-UCBD.<br>The grid cells are not always squared, as they deviate from a perfect rectangular 1x1 km shape depending on latitude and longitude of each grid cell.<br>The shape distortion adds uncertainty to our results for a very small number of urban centers.<br>These are located in very low and very high latitudes which are also far away from the Greenwich meridian, e.g. in New Zealand.<br>For each of the resulting 665,641 grid cells, we aggregated both the reference data sets (if available) and the datasets utilized as predictors in the model.<br>Potentially, a few urban centers might have been missed in the data set.<br>However, the data set should capture at least 99% of all urban centers at global scale.
Sampling strategy	This dataset does not represent a sample, but the whole population of urban centers globally.
Data collection	OSM uses a similar data collection approach to Wikipedia.<br>Registered users can contribute data to OSM via various editors on Desktop PCs (e.g. iD, JOSM) or using mobile applications (e.g. StreetComplete, GoMap!).<br>Currently there are about 10.3 Million registered OSM users.<br>Anonymized information about the OSM users is available in the OSM history database.
Timing and spatial scale	We included all data which have been mapped in OSM between 2008-01-01 (start date) and 2023-01-01 (stop date) on a global scale.
Data exclusions	No data have been excluded.
Reproducibility	Code and data has been provided in GitHub and on Figshare to reproduce our findings
Randomization	We did not collect a sample but used the whole population.<br>As such randomization was not applicable in this setting.<br>Data was used as it was available in OSM and the other datasets, no modifications or sampling were performed from our side.
Blinding	We did not perform an experiment which involved treatment.<br>There is no bias that could be cured by blinding in our procedure.<br>Our data represents the whole population and data were used as they were available without further modification or sampling.

Did the study involve field work? ☐ Yes ☒ No

Field work, collection and transport

Field conditions	not applicable
Location	not applicable
Access & import/export	not applicable
Disturbance	not applicable

Reporting for specific materials, systems and methods

We require information from authors about some types of materials, experimental systems and methods used in many studies. Here, indicate whether each material, system or method listed is relevant to your study. If you are not sure if a list item applies to your research, read the appropriate section before selecting a response.

Materials & experimental systems

n/a	Involved in the study
☒	☐ Antibodies
☒	☐ Eukaryotic cell lines
☒	☐ Palaeontology and archaeology
☒	☐ Animals and other organisms
☒	☐ Clinical data
☒	☐ Dual use research of concern

Methods

n/a	Involved in the study
☒	☐ ChIP-seq
☒	☐ Flow cytometry
☒	☐ MRI-based neuroimaging

Antibodies

Antibodies used	not applicable
Validation	not applicable

Eukaryotic cell lines

Policy information about cell lines and Sex and Gender in Research

Cell line source(s)	not applicable
Authentication	not applicable
Mycoplasma contamination	not applicable
Commonly misidentified lines (See ICLAC register)	not applicable

Palaeontology and Archaeology

Specimen provenance	not applicable
Specimen deposition	not applicable
Dating methods	not applicable

☐ Tick this box to confirm that the raw and calibrated dates are available in the paper or in Supplementary Information.

Ethics oversight	not applicable

Note that full information on the approval of the study protocol must also be provided in the manuscript.

Animals and other research organisms

Policy information about studies involving animals; ARRIVE guidelines recommended for reporting animal research, and Sex and Gender in Research

Laboratory animals	not applicable
Wild animals	not applicable
Reporting on sex	not applicable
Field-collected samples	not applicable
Ethics oversight	not applicable

Note that full information on the approval of the study protocol must also be provided in the manuscript.

Clinical data

Policy information about clinical studies

All manuscripts should comply with the ICMJE guidelines for publication of clinical research and a completed CONSORT checklist must be included with all submissions.

Clinical trial registration	not applicable
Study protocol	not applicable
Data collection	not applicable
Outcomes	not applicable

Dual use research of concern

Policy information about dual use research of concern

Hazards

Could the accidental, deliberate or reckless misuse of agents or technologies generated in the work, or the application of information presented in the manuscript, pose a threat to:

No	Yes	
☒	☐	Public health
☒	☐	National security
☒	☐	Crops and/or livestock
☒	☐	Ecosystems
☒	☐	Any other significant area

Experiments of concern

Does the work involve any of these experiments of concern:

No	Yes	
☒	☐	Demonstrate how to render a vaccine ineffective
☒	☐	Confer resistance to therapeutically useful antibiotics or antiviral agents
☒	☐	Enhance the virulence of a pathogen or render a nonpathogen virulent
☒	☐	Increase transmissibility of a pathogen
☒	☐	Alter the host range of a pathogen
☒	☐	Enable evasion of diagnostic/detection modalities
☒	☐	Enable the weaponization of a biological agent or toxin
☒	☐	Any other potentially harmful combination of experiments and agents

ChIP-seq

Data deposition

- [ ] Confirm that both raw and final processed data have been deposited in a public database such as GEO.
- [ ] Confirm that you have deposited or provided access to graph files (e.g. BED files) for the called peaks.

Data access links<br>*May remain private before publication.*	not applicable
Files in database submission	not applicable
Genome browser session<br>(e.g. UCSC)	not applicable

Methodology

Replicates	not applicable
Sequencing depth	not applicable
Antibodies	not applicable
Peak calling parameters	not applicable
Data quality	not applicable
Software	not applicable

Flow Cytometry

Plots

Confirm that:

- [ ] The axis labels state the marker and fluorochrome used (e.g. CD4-FITC).
- [ ] The axis scales are clearly visible. Include numbers along axes only for bottom left plot of group (a 'group' is an analysis of identical markers).
- [ ] All plots are contour plots with outliers or pseudocolor plots.
- [ ] A numerical value for number of cells or percentage (with statistics) is provided.

Methodology

Sample preparation	not applicable
Instrument	not applicable
Software	not applicable
Cell population abundance	not applicable
Gating strategy	not applicable

- [ ] Tick this box to confirm that a figure exemplifying the gating strategy is provided in the Supplementary Information.

Magnetic resonance imaging

Experimental design

Design type	not applicable
Design specifications	not applicable
Behavioral performance measures	not applicable

Acquisition

Imaging type(s)	not applicable
Field strength	not applicable
Sequence & imaging parameters	not applicable
Area of acquisition	not applicable

Diffusion MRI ☐ Used ☐ Not used

Preprocessing

Preprocessing software	not applicable
Normalization	not applicable
Normalization template	not applicable
Noise and artifact removal	not applicable
Volume censoring	not applicable

Statistical modeling & inference

Model type and settings	not applicable
Effect(s) tested	not applicable

Specify type of analysis: ☐ Whole brain ☐ ROI-based ☐ Both

Statistic type for inference (See Eklund et al. 2016)	not applicable
Correction	not applicable

Models & analysis

n/a	Involved in the study
☐	☐ Functional and/or effective connectivity
☐	☐ Graph analysis
☐	☐ Multivariate modeling or predictive analysis

Functional and/or effective connectivity	not applicable
Graph analysis	not applicable
Multivariate modeling and predictive analysis	not applicable
